# Early rheumatoid arthritis is characterized by a distinct and transient synovial fluid cytokine profile of T cell and stromal cell origin

**DOI:** 10.1186/ar1733

**Published:** 2005-04-07

**Authors:** Karim Raza, Francesco Falciani, S John Curnow, Emma J Ross, Chi-Yeung Lee, Arne N Akbar, Janet M Lord, Caroline Gordon, Christopher D Buckley, Mike Salmon

**Affiliations:** 1 MRC Centre for Immune Regulation, Division of Immunity and Infection, The University of Birmingham, Birmingham, UK; 2 Department of Rheumatology, City Hospital, Sandwell and West Birmingham Hospitals NHS Trust, Birmingham, UK; 3 School of Biosciences, The University of Birmingham, Birmingham, UK; 4 Department of Radiology, City Hospital, Sandwell and West Birmingham Hospitals NHS Trust, Birmingham, UK; 5 Department of Immunology and Molecular Pathology, Royal Free and University College Medical School, London, UK

## Abstract

Pathological processes involved in the initiation of rheumatoid synovitis remain unclear. We undertook the present study to identify immune and stromal processes that are present soon after the clinical onset of rheumatoid arthritis (RA) by assessing a panel of T cell, macrophage, and stromal cell related cytokines and chemokines in the synovial fluid of patients with early synovitis. Synovial fluid was aspirated from inflamed joints of patients with inflammatory arthritis of duration 3 months or less, whose outcomes were subsequently determined by follow up. For comparison, synovial fluid was aspirated from patients with acute crystal arthritis, established RA and osteoarthritis. Rheumatoid factor activity was blocked in the synovial fluid samples, and a panel of 23 cytokines and chemokines measured using a multiplex based system. Patients with early inflammatory arthritis who subsequently developed RA had a distinct but transient synovial fluid cytokine profile. The levels of a range of T cell, macrophage and stromal cell related cytokines (e.g. IL-2, IL-4, IL-13, IL-17, IL-15, basic fibroblast growth factor and epidermal growth factor) were significantly elevated in these patients within 3 months after symptom onset, as compared with early arthritis patients who did not develop RA. In addition, this profile was no longer present in established RA. In contrast, patients with non-rheumatoid persistent synovitis exhibited elevated levels of interferon-γ at initiation. Early synovitis destined to develop into RA is thus characterized by a distinct and transient synovial fluid cytokine profile. The cytokines present in the early rheumatoid lesion suggest that this response is likely to influence the microenvironment required for persistent RA.

## Introduction

The synovium is the primary site of pathology in rheumatoid arthritis (RA). The rheumatoid synovium contains large numbers of CD4^+ ^T cells. Patients with severe disease frequently express DR4 molecules that share an epitope in the third hypervariable region of the β-chain [1], suggesting a pathogenic role for T cells. However, the presence of only low levels of T cell related cytokines in the synovium and synovial fluid of established RA patients [2,3] led many to question the role of T cells in persistent disease. Nevertheless, this synovial cytokine profile is consistent with the highly differentiated CD45RO^bright^RB^dull ^phenotype of synovial T cells [4]. A widely accepted model has emerged in which the persistence of inflammation in established RA is driven by interactions between T cells, macrophages and fibroblasts in an abnormal microenvironment [5,6]. The synovial T cell population is maintained through active inhibition of apoptosis, mediated at least in part by fibroblast and macrophage derived type 1 IFNs, and active retention facilitated by fibroblast derived transforming growth factor-β [7-9]. Contact dependent interactions between T cells and macrophages stimulate the production of proinflammatory cytokines, including tumour necrosis factor (TNF)-α, in an antigen independent manner [10-12].

This model of persistence in established disease requires the presence of hyperplastic synovial tissue, which is unlikely to be present at the onset of RA. Consequently, the processes manifest at initiation that lead to persistence are likely to be distinct. Difficulties in accessing patients with very early disease and in sampling those joints involved at clinical onset have proved to be obstacles to addressing these issues. The role of T cells and antigen in the initiation of RA, the mechanisms that drive early fibroblast expansion, and the interplay between T cells and the stromal environment therefore remain obscure.

In order to study mechanisms of very early synovitis potentially leading to RA, we established a rapid access clinic with a wide recruitment base in which patients with synovitis were seen within the first few weeks after symptom onset. Using a multiplex bead based system, allowing simultaneous analysis of over 20 soluble molecules in very small sample volumes [13], we measured a panel of cytokines and chemokines in the synovial fluid of patients with early inflammatory arthritis. Patients whose disease subsequently fulfilled American Rheumatism Association (ARA) criteria for RA had a cytokine profile characterized by a range of T cell, stromal cell and macrophage related cytokines that was not present in long-standing RA. This profile was not seen in patients with other early arthritides. A model was built incorporating these cytokines that distinguished patients who progressed to RA from other early arthritis patients with a high degree of accuracy. These data suggest that the pathological mechanisms operating at the onset of clinically apparent RA are distinct from those in other early inflammatory arthritides, and that these mechanisms are transient. In addition, the present study supports the concept that T cells play a role in disease initiation that is different from their role in maintaining persistent inflammation.

## Materials and methods

### Patients

Patients were recruited through the rapid access clinic for early inflammatory arthritis at City Hospital, Birmingham, UK. Permission was obtained from the local ethics committee and all patients gave written informed consent. All patients had one or more swollen joints and symptoms (inflammatory joint pain and/or early morning stiffness and/or joint related soft tissue swelling) of duration 3 months or less. Patients with evidence of previous inflammatory joint disease were excluded. Joints were aspirated under either palpation or ultrasound guidance. Where the synovial fluid volume in the inflamed joint was very small (commonly at proximal interphalyngeal, metacarpophalyngeal and wrist joints) direct aspiration was not possible. Although the cellular content of these joints could be sampled using ultrasound guided lavage [14], lavage samples were excluded from this study because the potentially variable dilution made comparisons of the absolute levels of chemokines and cytokines unreliable. Synovial fluid was directly aspirated from the joints of 36 patients with non-crystal-related very early inflammatory arthritis. In addition, for comparison, synovial fluid was obtained from patients in three well defined diagnostic groups: early inflammatory arthritis of crystal origin that resolved (gout [*n *= 12] and pseudogout [*n *= 2]), established RA (*n *= 9) and osteoarthritis (*n *= 4). Synovial fluid was collected into nonheparinized tubes and spun at 1000 *g *for 10 min within 30 min of sample collection. The acellular portion of synovial fluid was stored at -70°C before subsequent analysis.

The 36 patients with early inflammatory arthritis were followed for 18 months and then assigned to their final diagnostic groups. Patients were classified as having RA according to the 1987 ARA criteria [15], allowing criteria to be satisfied cumulatively. Although the 1987 ARA criteria have no exclusions, we excluded from the RA category patients with alternative rheumatological diagnoses explaining their inflammatory arthritis. Thus, one patient, with polymyositis related arthritis, who fulfilled criteria for RA was excluded from the RA group and included in the non-rheumatoid persistent group. In addition, one patient, who fulfilled criteria for RA at presentation (but was seronegative for rheumatoid factor [RF] and anti-cyclic citrullinated peptide [CCP] antibody), had transient disease, remained symptom free and off all mediation at follow up, and was included in the resolving group. Patients were diagnosed with reactive arthritis (ReA), psoriatic arthritis (PsA) and a number of miscellaneous conditions according to established criteria. Of the 36 patients with non-crystal-related early inflammatory arthritis, 14 had a resolving disease (sexually acquired ReA [*n *= 4] and unclassified arthritis [*n *= 10]) and 22 developed persistent inflammatory arthritis (RA [*n *= 8], unclassified arthritis [*n *= 9], PsA [*n *= 2] and arthritis related to ulcerative colitis [*n *= 1], polymyositis [*n *= 1] and Behçet's disease [*n *= 1]). Three of these RA patients presented with inflammatory arthritis of just the knee or ankle joint(s) though polyarticular synovitis, including involvement of the small joints of the hands, developed subsequently.

### Anti-CCP antibody and rheumatoid factor assay

IgG anti-CCP antibody was detected using the DIASTAT™ anti-CCP assay (Axis-Shield Diagnostics Ltd., Dundee, UK) and seropositivity was defined as a titre of ≥5 IU/ml. RF was measured using an ELISA (AUTOSTAT™II Total RF; Hycor Biomedical Ltd., Penicuik, UK).

### Cytokine and chemokine assay

Twenty-three cytokines and chemokines were measured simultaneously in synovial fluid samples using a multiplex detection kit (Biosource International, Camarillo, CA, USA): IL-1β, IL-2, IL-4, IL-5, IL-6, IL-8, IL-10, IL-12, IL-13, IL-15, IL-17, macrophage inflammatory protein (MIP)-1α, MIP-1β, monocyte chemoattractant protein (MCP)-1, RANTES (regulated on activation, normal T expressed and secreted), eotaxin, TNF-α, IFN-γ, granulocyte–macrophage colony-stimulating factor (GM-CSF), granulocyte colony-stimulating factor, epidermal growth factor (EGF), basic fibroblast growth factor (bFGF) and vascular endothelial growth factor. A volume of 50 μl synovial fluid or of the cytokine/chemokine standards was preincubated with 50 μl blocking buffer (40% normal mouse serum [Sigma, Poole, UK], 20% goat serum [DakoCytomation Ltd, Ely, UK] and 20% rabbit serum [DakoCytomation Ltd]; see below) for 30 min. A volume of 50 μl diluted sample, or blocking buffer alone, was incubated with 25 μl of multiplex microspheres for 2 hours. Microspheres were washed with phosphate-buffered saline/0.05% Tween 20 and incubated with 25 μl of biotinylated detection antibody, diluted in 25 μl blocking buffer and 50 μl assay buffer (1% bovine serum albumin [Sigma] in phosphate-buffered saline/0.05% Tween 20), for 1 hour. Microspheres were then washed, incubated with streptavidin–PE at room temperature for 30 min and washed again. Subsequently, the microspheres were resuspended in 100 μl assay buffer and analyzed (Luminex^100 ^LabMAP™ system; Luminex Corporation, Austin, TX, USA). Cytokine and chemokine concentrations were calculated by reference to the standard curve.

False-positive results, caused by cross-linking of capture and detection antibodies by RF, are a common problem with any immunoassay of rheumatoid samples. We tested several strategies to eliminate this effect, including the absorption of RF, and found the optimal approach in this system to be blocking with a combination of 20% normal mouse serum, 10% rabbit serum and 10% goat serum. The validity of the results were assessed in two ways. First, a negative control microsphere was produced by conjugating a microsphere, with a different intrinsic fluorescence to any of those in the assay, to total mouse immunoglobulin (the capture antibodies on the microspheres were all mouse monoclonal antibodies). These microspheres were tested in parallel with those conjugated to specific antibodies. Second, an additional aliquot of each synovial fluid sample was tested with an entirely different detection kit obtained from an alternative source (Upstate Biotechnology, Milton Keynes, UK). This assay uses different antibody combinations to detect 14 of the cytokines and chemokines measured by the primary assay (IL-1β, IL-2, IL-4, IL-5, IL-6, IL-8, IL-10, IL-12, IL-13, MCP-1, RANTES, eotaxin, TNF-α and IFN-γ).

### Analysis

In order to identify cytokines that distinguish patients with early RA from other patients with early synovitis and from patients with established RA, we performed univariate and multivariate analyses. The Wilcoxon rank sum test was used for univariate analysis. The false discovery rate correction was used to correct for the multiple comparisons made [16]. Results are reported as *Q *values, which represent the likelihood of obtaining a given *P *value by chance given the multiple tests performed. We used a 1% false discovery rate (*Q *< 0.01) as our threshold for statistical significance.

Univariate statistical tests can be used to identify cytokines that are differentially expressed between two or more groups of patients. However, such analyses do not address the relative significance of different combinations of cytokines required to discriminate between different conditions. These discriminatory profiles are likely to provide insights to the biological processes underlying the different disease states. To identify groups of cytokines that allow the distinction of potential outcomes in patients with early arthritis, we used a classification algorithm termed Random Forest [17,18]. This method is based on the principal of decision trees and incorporates efficient methods to establish the importance of each cytokine in the classification and to perform an unbiased estimate of classification error. Random Forest does not use cross-validation or a separate training set to obtain an unbiased estimate of classification error. Instead, a collection of decision trees is constructed using a different randomly chosen (bootstrap) sample of two-thirds of the data for each tree. Each branching point, or node, is produced by using the best of a subset of predictors randomly chosen at that node. One-third of the cases are left out of each bootstrap sample and not used in the construction of any given decision tree. Each of these cases left out in the construction of a particular tree is then put back into that tree to test the validity of the classification obtained from the bootstrap sample. In this way, a test set classification is obtained for each case, in about one-third of the trees. The classification error is defined as the proportion of patients who are misclassified. This strategy has previously been shown to perform well compared with other classifiers, including discriminant analysis, support vector machines and neural networks; it is also robust against overfitting [17].

The Random Forest algorithm also estimates the relative importance of each variable in contributing to the classification process. In this study we used Random Forest to rank the contribution of each cytokine to discrimination between outcomes of patients with early synovitis, as a possible index of their biological contribution to this discrimination. This analysis does not emphasize cytokines that are of general importance to inflammation.

The models developed using Random Forest can be visualized graphically by using multidimensional scaling to plot the relative similarity between patients in the trees [19]. This allows the magnitude of the difference between groups, and the utility of the data set in distinguishing different outcomes, to be assessed.

## Results

### Patient characteristics

Baseline characteristics of patients with early inflammatory arthritis of non-crystal origin are shown in Table 1. Patients who developed RA were significantly older than patients in other groups. There were no significant differences in symptom duration or C-reactive protein (CRP) level at initial presentation between the groups. Of the nine patients with established RA, four were female and the median age was 62 years (interquartile range 43–74 years). There were no significant sex or age differences between the established RA and early RA patients.

### Cytokine and chemokine levels

**Figure 1:**
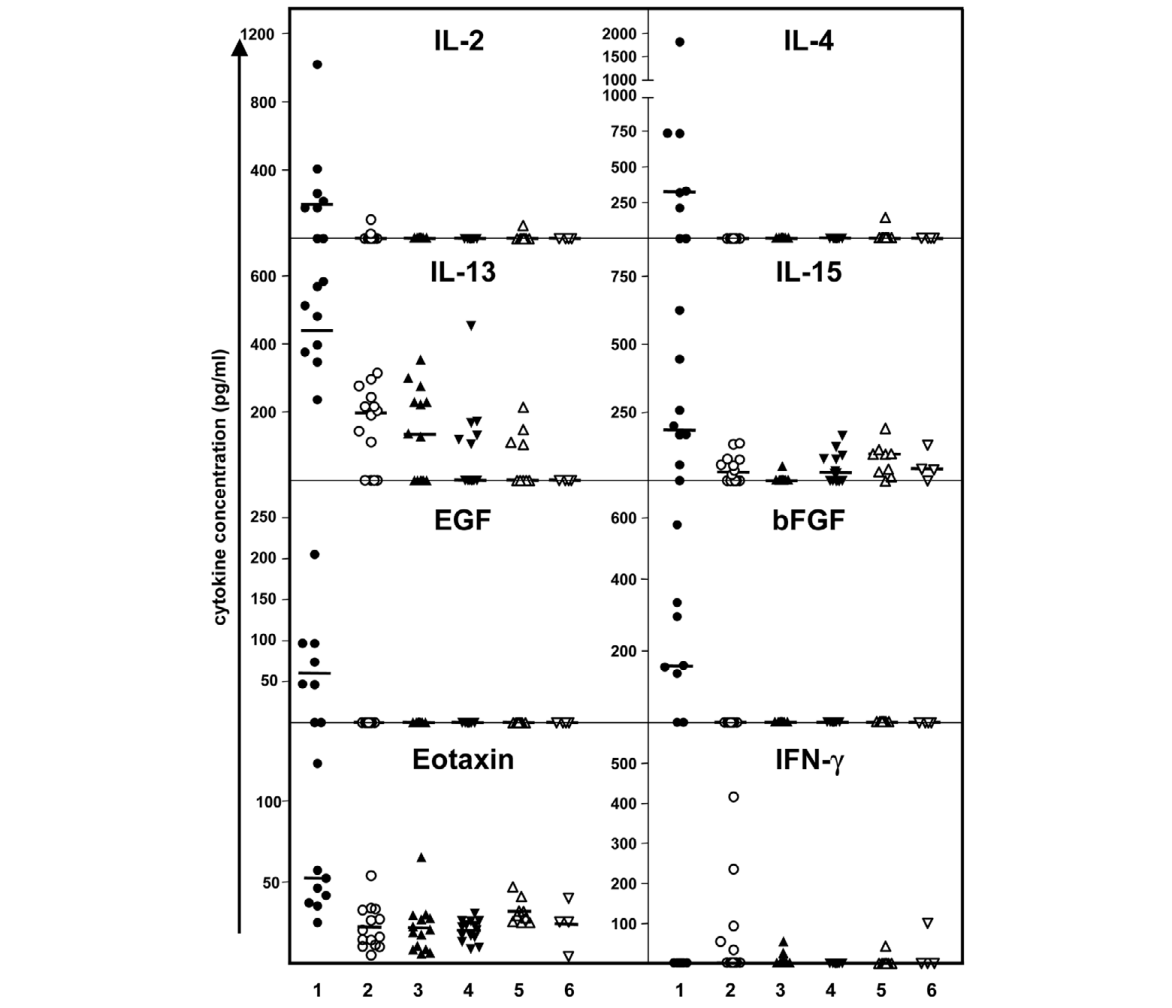
Synovial fluid cytokines in early and established arthritis. Shown are synovial fluid concentrations (pg/ml) of IL-2, IL-4, IL-13, IL-15, epidermal growth factor (EGF), basic fibroblast growth factor (bFGF), eotaxin and IFN-γ. Patient groups: 1, early synovitis that develops into rheumatoid arthritis (RA); 2, early synovitis that develops into non-rheumatoid persistent synovitis; 3, early non-crystal-related resolving synovitis; 4, crystal-related resolving synovitis; 5, established RA; and 6, osteoarthritis.

**Figure 2:**
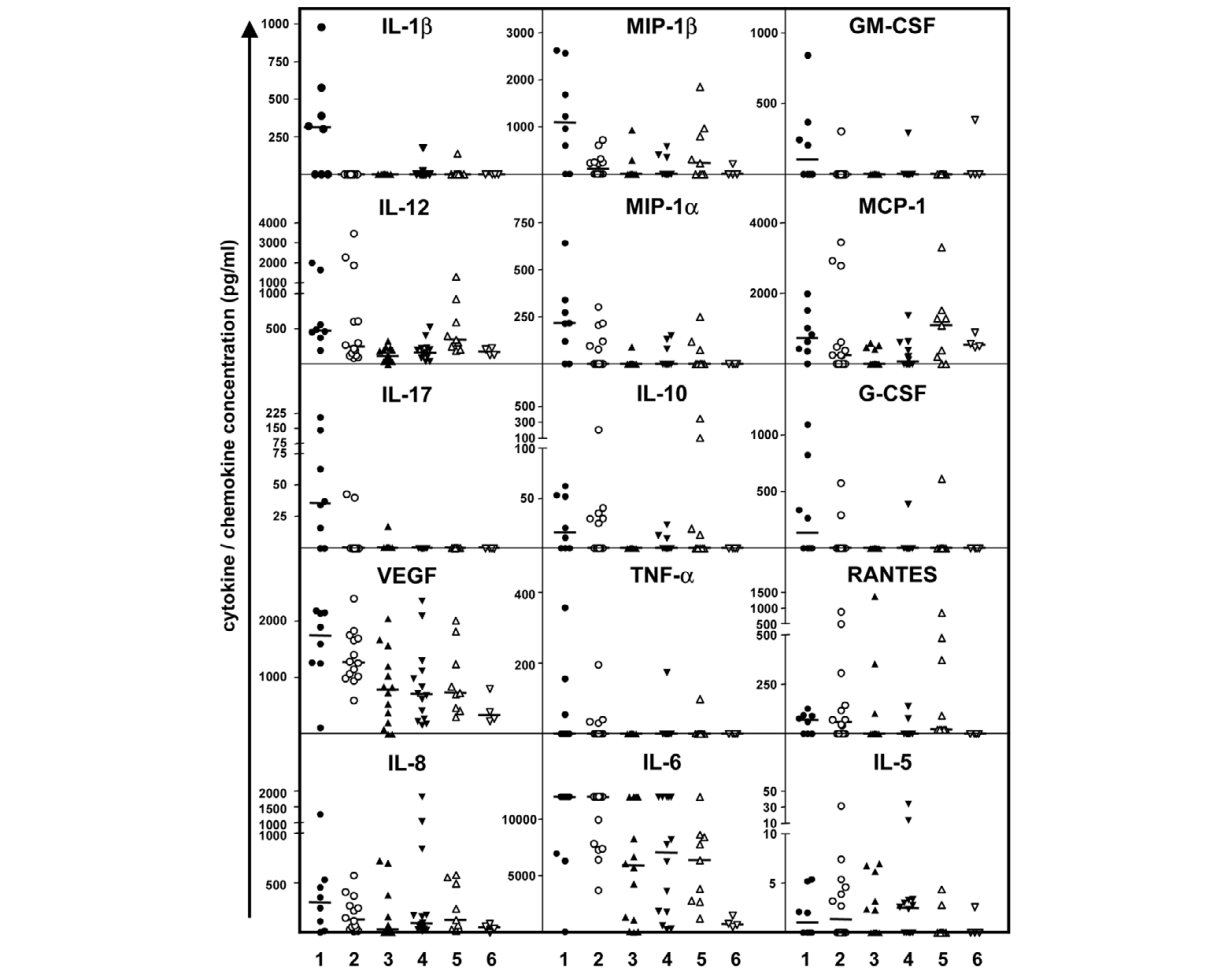
Synovial fluid cytokines in early and established arthritis. Shown are synovial fluid concentrations (pg/ml) of IL-1β, macrophage inflammatory protein (MIP)-1β, granulocyte–macrophage colony-stimulating factor (GM-CSF), IL-12, MIP-1α, monocyte chemoattractant protein (MCP)-1, IL-17, IL-10, granulocyte colony-stimulating factor (G-CSF), vascular endothelial growth factor (VEGF), tumour necrosis factor (TNF)-α, RANTES (regulated on activation, normal T expressed and secreted), IL-8, IL-6 and IL-5. Patient groups: 1, early synovitis that develops into rheumatoid arthritis (RA); 2, early synovitis that develops into non-rheumatoid persistent synovitis; 3, early non-crystal-related resolving synovitis; 4, crystal-related resolving synovitis; 5, established RA; and 6, osteoarthritis.

The levels of synovial cytokines in patients with early and established synovitis are shown in Figs 1 and 2, and the statistical significance of differences between cytokine levels in patient groups is shown in Table 2. Patients with early synovitis destined to develop RA exhibited a cytokine profile in synovial fluid that was distinct from that of patients with other early inflammatory arthritides (early synovitis that developed into non-rheumatoid persistent disease plus non-crystal-related resolving arthritis plus crystal-related resolving arthritis). Early RA synovial fluid was characterized by significantly elevated levels of T cell related cytokines (IL-2, IL-4, IL-13 and IL-17) and stromal cell and macrophage related cytokines (EGF, bFGF, IL-1 and IL-15) when compared with synovial fluid from patients with other early synovitis (Figs 1 and 2, and Table 2). Although levels of the Th2-type cytokines IL-4 and IL-13 were elevated in patients with early RA, IFN-γ was never detected in these patients (Fig. 1). In contrast, IFN-γ was detected in five patients with early non-rheumatoid persistent disease (one PsA, one ulcerative colitis related arthritis and three unclassified) and in three patients with early self-limiting disease (two ReA and one unclassified; Fig. 1). In addition, the synovial cytokine profile of patients with early synovitis destined to develop RA was significantly different from that of patients with established RA. Patients with early RA had significantly elevated synovial levels of IL-2, IL-4, IL-13, IL-17, EGF and bFGF when compared with patients with established RA (Figs 1 and 2, and Table 2).

**Figure 3:**
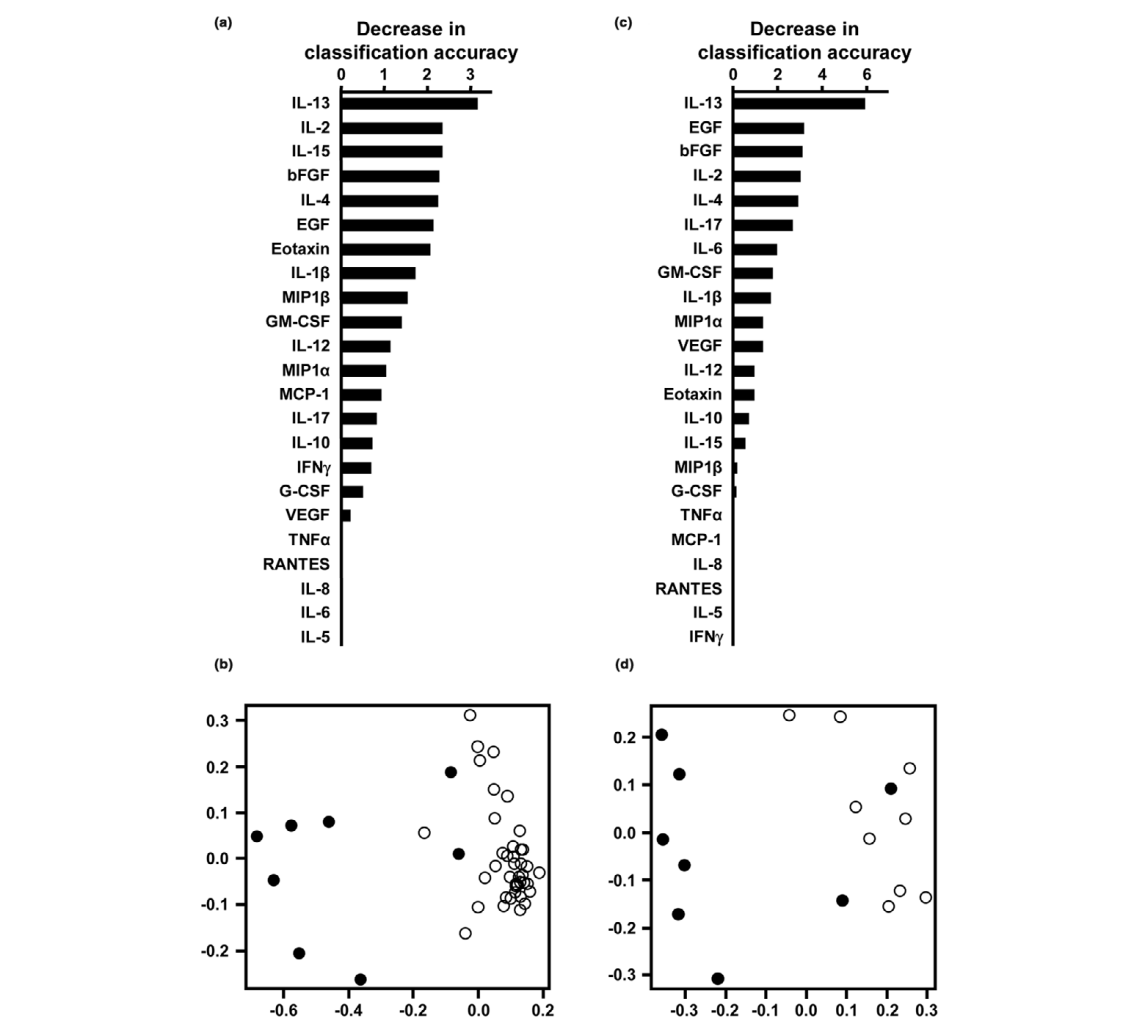
Importance of individual cytokines in classifying patient groups. Plots represent models discriminating patients with early inflammatory arthritis who develop rheumatoid arthritis (RA) from **(a,b) **all other early inflammatory arthritis patients (early disease that progresses to non-rheumatoid persistent disease plus early non-crystal-related resolving arthritis plus crystal-related resolving arthritis) and from **(c,d) **established RA. Panels **a **and **c **show the relative importance of the cytokines in the overall classification. The vertical axes represent individual cytokines arranged according to importance. The horizontal axes represent the average decrease in classification accuracy seen when the values for each cytokine are permuted. Important cytokines are associated with a greater decrease in classification accuracy. The plots shown in panels **b **and **d **are metric multidimensional scaling (MDS) representations of the proximity matrices of the Random Forest models demonstrating the relationship between individual patients in the two models. The two axes represent the first and second MDS axes. Closed circles represent patients with early inflammatory arthritis who develop RA in both panels; open circles represent patients with (panel **b**) all other early inflammatory arthritis and (panel **d**) established RA.

We assessed the relative importance of the cytokines measured in distinguishing patients with early disease destined to develop RA from patients with all other early arthritis together (early disease that progressed to non-rheumatoid persistent disease plus non-crystal-related resolving arthritis plus crystal-related resolving arthritis) and patients with established RA. The relative importance of each cytokine in the accuracy of classification in these models is shown in Fig. 3a,c. IL-13, together with IL-2, IL-4, IL-15, bFGF and EGF, were the most important cytokines in distinguishing early RA patients from other patients with early synovitis, with high levels of these cytokines predicting the development of RA. Similarly, IL-13, together with IL-2, IL-4, IL-17, bFGF and EGF, were the most important cytokines in distinguishing early RA patients from patients with established RA. Using this approach two of the eight early RA patients were misclassified in both models. One of these was an 82-year-old man who presented with a 6-week history of synovitis (RF negative, anti-CCP antibody negative, CRP 51 mg/l); the other was a 70-year-old woman who presented with a 10-week history of synovitis (RF positive, anti-CCP antibody positive, CRP 25 mg/l). The relationships between the different patients in the two models and the ability of the synovial cytokines to distinguish between different patient outcomes are shown in Fig. 3b,d.

**Figure 4:**
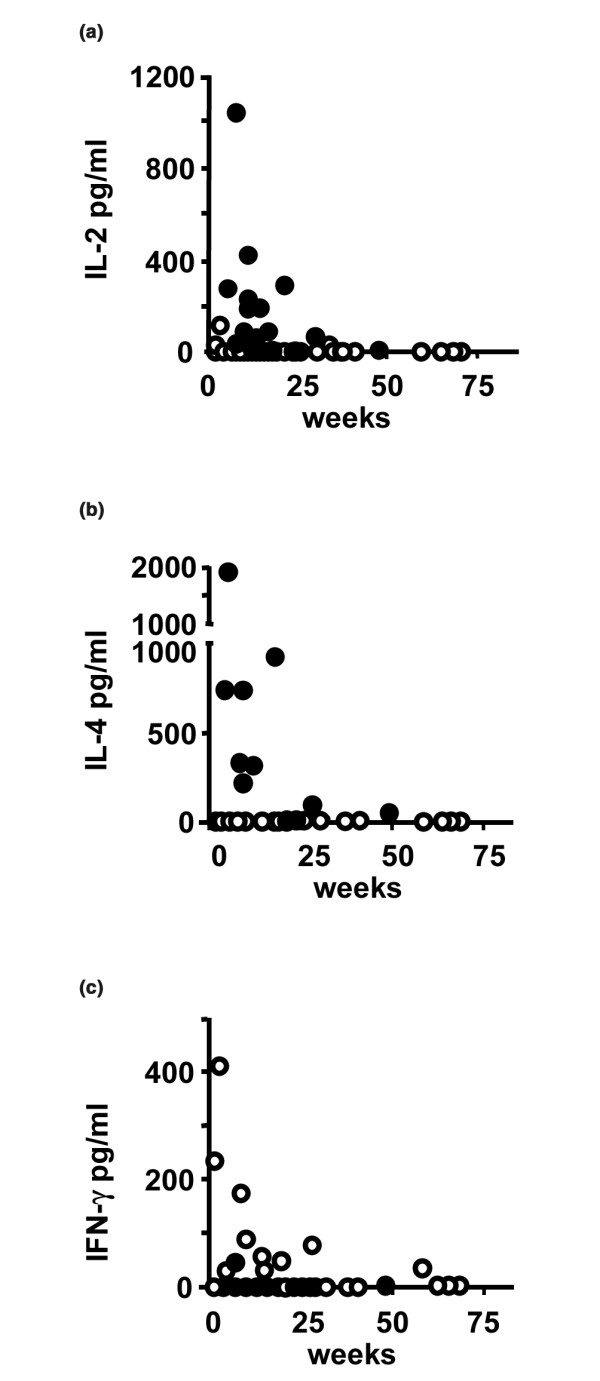
Longitudinal synovial fluid cytokine concentrations (pg/ml) in patients with early inflammatory arthritis. Results are shown for patients with early inflammatory arthritis who develop rheumatoid arthritis (closed circles) and non-rheumatoid persistent inflammatory arthritis (open circles) for **(a) **IL-2, **(b) **IL-4 and **(c) **IFN-γ.

The cytokine profile that was seen in patients with early RA was transient. It was not seen in established RA (Figs 1 and 2) or after the first few months of symptoms in patients with early disease that went on to persist (Fig. 4). The transient nature of the elevations in IL-2 and IL-4 in early RA synovial fluid (Fig. 4) was also apparent for IL-13, IL-15, EGF and bFGF.

The validity of the results obtained using the multiplex assay was confirmed in two ways. First, no significant irrelevant staining was observed with any of the synovial fluid samples using the negative control microspheres. The median fluorescence intensity of the negative control microspheres when incubated with the synovial fluid samples was 14 (standard deviation 2.6) and when incubated with assay buffer alone was 16.3 (standard deviation 0.5). Second, the correlations between the results obtained using the two different antibody detection systems were all highly statistically significant and were as follows (expressed as Spearman's rank correlation [r_s_]): IL-1β, r_s _= 0.65 (*P *< 0.0001); IL-2, r_s _= 0.77 (*P *< 0.0001); IL-4, r_s _= 0.63 (*P *< 0.0001); IL-5, r_s _= 0.5 (*P *< 0.0001); IL-6, r_s _= 0.95 (*P *< 0.0001); IL-8, r_s _= 0.8 (*P *< 0.0001); IL-10, r_s _= 0.56 (*P *< 0.0001); IL-12, r_s _= 0.27 (*P *= 0.006); MCP-1, r_s _= 0.72 (*P *< 0.0001); RANTES, r_s _= 0.84 (*P *< 0.0001); TNF-α, r_s _= 0.47 (*P *< 0.0001); and IFN-γ, r_s _= 0.54 (*P *< 0.0001). Levels of eotaxin and IL-13 were below the detection limit of the second assay.

There was no significant correlation between the RF titre and the levels of any of the cytokines or chemokines measured in patients with early RA. Correlations between CRP and levels of chemokines and cytokines in early inflammatory arthritis synovial fluid were statistically significant only for IL-6 (r_s _= 0.49 [*P *= 0.003]), TNF-α (r_s _= 0.37 [*P *= 0.03]), IFN-γ (r_s _= 0.52 [*P *= 0.001]) and vascular endothelial growth factor (r_s _= 0.47 [*P *= 0.004]). These correlations were independent of outcome. An independent analysis of the eight patients with early RA revealed no correlation between CRP and the level of any chemokine or cytokine.

## Discussion

The synovium of patients with established RA is expanded and contains large numbers of fibroblasts, macrophages and highly differentiated T cells [20]. Although the mechanisms responsible for persistence of this infiltrate in established disease have been well characterized (for review [5,21,22]), those involved in disease initiation have not. A number of groups have studied RA at a relatively early stage from pathological, radiological and therapeutic perspectives. The maximum duration of symptoms accepted for recruitment to such studies has been highly variable, usually ranging from less than 1 year to less than 3 years [23-26]. However, the observation that spontaneous remission is unusual if inflammatory arthritis has persisted for longer than 6 months [27] suggests that pathological mechanisms driving the switch to persistence are already established by this time. In contrast, the concept that RA patients with a much shorter disease duration may be within a therapeutically distinct window is supported by a study in which remission was more commonly induced in patients with disease of ≤4 months duration compared with longer duration disease [28]. However, the confounding effect of differential rates of spontaneous remission has been difficult to exclude. Whether this very early phase of disease is pathologically distinct remains to be determined.

Within the first 12 weeks after symptom onset, we found that the synovial fluid of patients who eventually developed RA was characterized by a wide range of cytokines and chemokines. Some, such as IL-6, were present in all inflammatory arthritides, suggesting their importance in synovitis *per se *rather than a specific role in rheumatoid synovitis. In contrast, early RA patients had a distinct and consistent synovial cytokine profile characterized by T cell, macrophage and stromal cell related cytokines (in particular IL-2, IL-4, IL-13, IL-17, IL-1, IL-15, bFGF and EGF), which was not seen in other early arthritides. This profile was transient, and was no longer present in any patients with established RA. Seven of the eight patients whose disease developed into RA already expressed RF and anti-CCP antibody at this early stage, within weeks of symptom onset. Several groups have shown that these antibodies can be found in patients who subsequently develop RA, long before symptoms are apparent [29-31], implying a preclinical pathology. Our data are entirely compatible with the possibility of a preclinical phase of disease in patients with RA. We are clearly unable to address the issue of how the synovial cytokine profile within the first few months of symptoms compares with that which may be present during a preclinical phase of disease. However, our data do suggest that the pathological processes operating in the rheumatoid joint within the first few weeks after symptom onset differ from those processes operating in other early synovial lesions, and that these processes are transient.

In other studies comparing early RA (defined as a symptom duration of <1 year) and established RA, immunohistological analysis of the synovium, including an assessment of TNF-α, IL-1β and IL-6 expression, did not reveal any differences between early (mean disease duration 6 months) and long-standing disease [23]. Expression of IFN-γ, IL-10 and IL-12 mRNA in synovial fluid mononuclear cells were also similar between such early patients and those with established RA [32]. However, very few groups have studied the pathology of RA within the first few weeks of symptom onset. Indeed, because patients with a symptom duration of less than 6 weeks cannot fulfil 1987 ARA classification criteria for RA [15], any study of early disease that limits itself to patients fulfilling these criteria will exclude those with very early synovitis. We studied patients with inflammatory arthritis of duration 3 months or less, recruited through a rapid access clinic. The assignment of patients to specific diagnostic categories was done subsequently at clinical follow up. In one of the few other studies of the pathology of very early inflammatory arthritis, synovial histology was assessed in 24 patients with disease of duration under 2 months [33]. Nine patients had transient synovitis and six developed RA. Interestingly, there were no clear histological differences between these patients. In contrast, our results suggest that pathological mechanisms operating at the onset of clinically apparent RA are different from those in patients with other early arthritides. The results also show a surprising degree of uniformity between RA patients in this early phase of disease.

The concept that T cells play an important role in the initiation of RA, with antigen specific T cells mediating autoimmunity, is not new (for review [34]). However, robust evidence to support such a role for T cells has been lacking. The observation in this report of consistently elevated T cell derived cytokines strongly suggests T cell activity in early RA. The clear difference between the levels of specific T cell related cytokines in early RA and other early inflammatory arthritides, as well as established RA, suggests a pathological process that is specific to early RA and that is transient. The Th2 cytokine pattern, with a marked absence of IFN-γ and predominance of IL-4 and IL-13, in the earliest clinically apparent RA lesions was unexpected. Current paradigms suggest the cytokine balance in established RA to be skewed in favour of a Th1-type response [34]. In established RA, synovial T cells produce IFN-γ after *in vitro *stimulation [35], and mRNA for IFN-γ but not IL-4 is found in synovial fluid mononuclear cells [32]. However, recent evidence from mouse models suggests that IFN-γ plays a significant role in the resolution of synovial inflammation [36]. In murine models of *Leishmania *infection, a Th2 polarized response leads to persistent disease, whereas a Th1 polarized delayed-type hypersensitivity response leads to resolution [37]. This is similar to the effects seen in human leprosy [38]. The discrepancy between IFN-γ levels in early RA and other early arthritides may consequently be of relevance to the pathology of the transition to persistent inflammation. In addition, T cells cloned from early rheumatoid synovium have been shown to produce significantly more IL-4 than those from long-standing disease [39]. When T cells were re-cloned from an early patient at a subsequent time point, IL-4 production was significantly diminished [39]. These findings concur with the data reported here, suggesting a transient Th2 phenotype in early RA synovial fluid.

The role of the Th2-type cytokines in early RA is unclear. IL-4 has divergent proinflammatory and anti-inflammatory effects in animal models of inflammatory arthritis [40,41]. IL-13 induces proliferation and CD154 (CD40 ligand) expression in lung fibroblasts [42,43] and is important in inducing fibrosis in Th2 mediated diseases such as schistosomiasis [44]. In addition, both IL-4 and IL-13 protect synoviocytes against nitric oxide induced apoptosis [45]. The pro-survival and proliferative effects of these cytokines may be important in the development of the expanded fibroblast network, which occurs during early disease and which characterizes established RA [46]. The presence of significant levels of the autocrine synovial fibroblast growth factors bFGF and EGF [47] clearly supports this process. The absence of these growth factors in the synovial compartment of patients with self-limiting disease is not surprising, because one would not expect an expanded fibroblast layer (which would mediate the switch to persistence) in such patients. Interestingly, however, these growth factors were absent in non-RA persistent inflammatory arthritis, suggesting a difference between the mechanism of synovial hyperplasia in early RA and other persistent inflammatory arthritides.

Th2-type cytokines have additional effects on synovial fibroblasts that may be relevant in early RA. Cultured synovial fibroblasts have a global gene expression profile that is quite different from that of lymph node and tonsil fibroblasts [48]. However, the addition of IL-4 to synovial fibroblasts dramatically modulates their gene expression profile, which converges with that of fibroblasts from secondary lymphoid tissue [48]. Germinal centre-like structures are seen in the synovium of many RA patients [49]. The synovial environment in early RA may modulate fibroblast function, leading to the production of factors facilitating lymphoid aggregate formation and allowing local RF production.

The distinct T cell related cytokine profile observed in patients with early RA supports the concept that T cells play an important role at the onset of clinically apparent disease. Tissue dendritic cells (DCs) are specialized for high antigen capture and migration into draining lymph nodes, where they are essential for activating naïve T cells. The role played by DCs in the onset of inflammatory arthritis has been explored in a murine model, in which collagen pulsed mature DCs induced inflammatory arthritis when transferred into DBA/1 mice [50]. Characterization of the T cell response in draining lymph nodes revealed significant proliferation of collagen specific T cells and IL-2 production, suggesting that priming of autoreactive T cells by DCs may play a role in disease initiation. Blood monocytes may differentiate into DCs in the presence of GM-CSF, IL-4 and TNF-α [51] and early myeloid DC progenitors in RA synovial fluid differentiate in response to IL-4 or IL-13 in combination with GM-CSF and stem cell factor [52]. The synovial cytokine environment in early RA may thus stimulate mature DC production and consequent T cell activation.

The recruitment of leucocytes into the synovial compartment is regulated, in part, by chemokines. Although levels of the chemokines measured (e.g. MIP-1α, MIP-1β, MCP-1 and RANTES) were elevated in the synovial fluid of many patients with early RA, they were of limited value in distinguishing early RA from other early arthritides or from established RA. The chemokine stromal cell-derived factor 1 (SDF-1; CXC chemokine ligand 12 [CXCL12]) has been implicated in the recruitment and retention of T cells and monocytes into the rheumatoid synovium [9,53]. Unfortunately, no combination of monoclonal antibodies could be found that produced reliable estimates of SDF-1 using the multiplex detection strategy, so we were unable to measure this chemokine.

Several roles can be proposed for the other cytokines found in early RA. IL-17 has pleiotropic effects on leucocytes and stromal cells (for review [54]). For example, it induces IL-6 and IL-8 production by fibroblasts [55] and stimulates macrophage IL-1 and TNF-α production [56]. IL-15 stimulated T cells induce macrophage-mediated TNF-α production [11]. In addition, all common γ-chain cytokines, including IL-2, IL-4 and IL-15, are potent T cell survival factors [57], which may support the persistence of the early rheumatoid lesion.

## Conclusion

The data presented herein suggest that the pathology of RA within the first few months after symptom onset is distinct from that of other early inflammatory arthritides and of established RA. The nature of the cytokines present in the synovial fluid of patients with early RA suggests that this response is likely to influence the microenvironment required for persistent disease.

## Abbreviations

ARA = American Rheumatism Association; bFGF = basic fibroblast growth factor; CCP = cyclic citrullinated peptide; CRP = C-reactive protein; DC = dendritic cell; EGF = epidermal growth factor; GM-CSF = granulocyte–macrophage colony-stimulating factor; IFN = interferon; IL = interleukin; MCP = monocyte chemoattractant protein; MIP = macrophage inflammatory protein; PsA = psoriatic arthritis; RA = rheumatoid arthritis; RANTES = regulated on activation, normal T expressed and secreted; ReA = reactive arthritis; RF = rheumatoid factor; Th = T-helper (cell); TNF = tumour necrosis factor.

## Competing interests

The author(s) declare that they have no competing interests.

## Authors' contributions

KR participated in the design of the study, recruited and followed up the early arthritis patients, analyzed and interpreted the data, and drafted the manuscript. FF performed the statistical analysis and was involved in drafting the manuscript. SJC acquired the cytokine and chemokine data, analyzed and interpreted the data, and was involved in drafting the manuscript. ET acquired the cytokine and chemokine data. CYL participated in assessing patients and in performing ultrasound guided joint aspirations. ANA, JML, CG and CB participated in the design of the study and interpretation of data. MS participated in the design of the study and interpretation of data, and was involved in drafting the manuscript. All authors have read and approved the final manuscript.

## Figures and Tables

**Table 1:** Baseline characteristics of patients with early inflammatory arthritis of non-crystal origin

	RA	Non-RA persistent	Resolving	*P*
Number	8	14	14	
Female (*n*)	4	6	6	NS^a^
Age (years)^b,c^	65.5 (55–73)	29.5 (23.5–61)	28.5 (21–45)	0.006^d^ RA versus non-RA persistent <0.05^e^ RA versus resolving <0.01^e^
Symptom duration (weeks)^b,c^	9 (6–9.5)	7.5 (2.5–11.5)	4 (2–8)	NS^d^
CRP (mg/l)^b,c^	31 (27.5–52.5)	67 (29–172.5)	24 (3.5–45)	NS^d^
RF ≥30 IU/ml (*n*)	7	0	2	<0.0001^a^
Anti-CCP antibodies ≥5 IU/ml (*n*)	7	0	1	<0.0001^a^
RF ≥30 IU/ml + Anti-CCP antibodies ≥5 IU/ml (*n*)	7	0	0	<0.0001^a^
Initial NSAID use (*n*)	7	6	8	NS^a^
Initial DMARD use (*n*)	0	0	0	NS^a^
Initial prednisolone use (*n*)	0	3	0	NS^a^
Joint aspirated (knee/ankle [*n*])	5/3	13/1	11/3	NS^a^

^a^χ^2 ^test. ^b^Median. ^c^Interquartile range. ^d^Kruskall–Wallis test. ^e^Where the medians were significantly different at the 5% level, Dunn's post-test was used to compare individual groups. CCP, cyclic citrullinated peptide; CRP, C-reactive protein; DMARD, disease-modifying antirheumatic drug; NS, not significant; NSAID, nonsteroidal antirheumatic drug; RA, rheumatoid arthritis; RF, rheumatoid factor.

**Table 2:** Comparisons between synovial fluid cytokineconcentrations in patients with early synovitis that develops into rheumatoid arthritis and other patient groups

Cytokine	Early RA versus all other early synovitis		Early RA versus established RA	
	
	*P*	*Q*	*P*	*Q*
IL-13	<0.00001	0.00002	0.00008	0.0004
EGF	<0.00001	0.00002	0.002	0.007
bFGF	<0.00001	0.00002	0.002	0.007
IL-4	<0.00001	0.00002	0.004	0.0097
IL-2	<0.00001	0.00002	0.004	0.0097
Eotaxin	0.00003	0.0002	NS	NS
IL-1	0.00003	0.0002	NS	NS
IL-17	0.00005	0.0003	0.002	0.007
IL-15	0.0001	0.0005	NS	NS
MIP-1β	0.0005	0.002	NS	NS
MIP-1α	0.0006	0.002	NS	NS
IL-12	0.002	0.006	NS	NS
GM-CSF	0.002	0.006	NS	NS
G-CSF	0.004	0.0097	NS	NS

Comparator groups were, first, patients with all other early synovitis (synovitis that develops into non-rheumatoid persistent synovitis plus non-crystal-related resolving synovitis plus crystal-related resolving synovitis) and, second, patients with established rheumatoid arthritis (RA). Groups were compared using the Wilcoxon rank sum test. The significance of the difference between groups is shown (*P*). The false discovery rate correction was used to correct for the multiple comparisons made. Results are reported as *Q *values, which represent the likelihood of obtaining a given *P *value by chance, given the multiple tests performed. For cytokines not shown there were no significant differences between groups (a 1% false discovery rate [*Q *< 0.01] was used as the threshold for statistical significance). bFGF, basic fibroblast growth factor; EGF, epidermal growth factor; G-CSF, granulocyte colony-stimulating factor; GM-CSF, granulocyte–macrophage colony-stimulating factor; IL, interleukin; MIP, macrophage inflammatory protein; NS, not significant.

## References

[B1] Gregersen PK, Silver J, Winchester RJ (1987). The shared epitope hypothesis. An approach to understanding the molecular genetics of susceptibility to rheumatoid arthritis. Arthritis Rheum.

[B2] Husby G, Williams RC (1985). Immunohistochemical studies of interleukin-2 and gamma-interferon in rheumatoid arthritis. Arthritis Rheum.

[B3] Firestein GS, Zvaifler NJ (1987). Peripheral blood and synovial fluid monocyte activation in inflammatory arthritis. II. Low levels of synovial fluid and synovial tissue interferon suggest that gamma-interferon is not the primary macrophage activating factor. Arthritis Rheum.

[B4] Matthews N, Emery P, Pilling D, Akbar A, Salmon M (1993). Subpopulations of primed T helper cells in rheumatoid arthritis. Arthritis Rheum.

[B5] Buckley CD, Pilling D, Lord JM, Akbar AN, Scheel-Toellner D, Salmon M (2001). Fibroblasts regulate the switch from acute resolving to chronic persistent inflammation. Trends Immunol.

[B6] Firestein GS, Zvaifler NJ (2002). How important are T cells in chronic rheumatoid synovitis?: II. T cell-independent mechanisms from beginning to end. Arthritis Rheum.

[B7] Salmon M, Scheel-Toellner D, Huissoon AP, Pilling D, Shamsadeen N, Hyde H, D'Angeac AD, Bacon PA, Emery P, Akbar AN (1997). Inhibition of T cell apoptosis in the rheumatoid synovium. J Clin Invest.

[B8] Pilling D, Akbar AN, Girdlestone J, Orteu CH, Borthwick NJ, Amft N, Scheel-Toellner D, Buckley CD, Salmon M (1999). Interferon-beta mediates stromal cell rescue of T cells from apoptosis. Eur J Immunol.

[B9] Buckley CD, Amft N, Bradfield PF, Pilling D, Ross E, Arenzana-Seisdedos F, Amara A, Curnow SJ, Lord JM, Scheel-Toellner D (2000). Persistent induction of the chemokine receptor CXCR4 by TGF-beta 1 on synovial T cells contributes to their accumulation within the rheumatoid synovium. J Immunol.

[B10] Sebbag M, Parry SL, Brennan FM, Feldmann M (1997). Cytokine stimulation of T lymphocytes regulates their capacity to induce monocyte production of tumor necrosis factor-alpha, but not interleukin-10: possible relevance to pathophysiology of rheumatoid arthritis. Eur J Immunol.

[B11] McInnes IB, Leung BP, Sturrock RD, Field M, Liew FY (1997). Interleukin-15 mediates T cell-dependent regulation of tumor necrosis factor-alpha production in rheumatoid arthritis. Nat Med.

[B12] Brennan FM, Hayes AL, Ciesielski CJ, Green P, Foxwell BM, Feldmann M (2002). Evidence that rheumatoid arthritis synovial T cells are similar to cytokine-activated T cells: involvement of phosphatidylinositol 3-kinase and nuclear factor kappaB pathways in tumor necrosis factor alpha production in rheumatoid arthritis. Arthritis Rheum.

[B13] Curnow SJ, Scheel-Toellner D, Jenkinson W, Raza K, Durrani OM, Faint JM, Rauz S, Wloka K, Pilling D, Rose-John S (2004). Inhibition of T cell apoptosis in the aqueous humor of patients with uveitis by IL-6/soluble IL-6 receptor trans-signaling. J Immunol.

[B14] Raza K, Lee CY, Pilling D, Heaton S, Situnayake RD, Carruthers DM, Buckley CD, Gordon C, Salmon M (2003). Ultrasound guidance allows accurate needle placement and aspiration from small joints in patients with early inflammatory arthritis. Rheumatology (Oxford).

[B15] Arnett FC, Edworthy SM, Bloch DA, McShane DJ, Fries JF, Cooper NS, Healey LA, Kaplan SR, Liang MH, Luthra HS (1988). The American Rheumatism Association 1987 revised criteria for the classification of rheumatoid arthritis. Arthritis Rheum.

[B16] Benjamini Y, Hochberg Y (1995). Controlling the false discovery rate: a practical and powerful approach to multiple testing. J R Stat Soc B.

[B17] Breiman L (2001). Random Forrests. Machine Learning.

[B18] Breiman L Manual on Setting Up, Using, and Understanding Random Forests v31.

[B19] Cox TF, Cox MAA (2000). Multidimensional scaling.

[B20] Zvaifler NJ (1973). The immunopathology of joint inflammation in rheumatoid arthritis. Adv Immunol.

[B21] Akbar AN, Salmon M (1997). Cellular environments and apoptosis: tissue microenvironments control activated T-cell death. Immunol Today.

[B22] Pap T, Muller-Ladner U, Gay RE, Gay S (2000). Fibroblast biology. Role of synovial fibroblasts in the pathogenesis of rheumatoid arthritis. Arthritis Res.

[B23] Tak PP, Smeets TJ, Daha MR, Kluin PM, Meijers KA, Brand R, Meinders AE, Breedveld FC (1997). Analysis of the synovial cell infiltrate in early rheumatoid synovial tissue in relation to local disease activity. Arthritis Rheum.

[B24] Conaghan PG, O'Connor P, McGonagle D, Astin P, Wakefield RJ, Gibbon WW, Quinn M, Karim Z, Green MJ, Proudman S (2003). Elucidation of the relationship between synovitis and bone damage: a randomized magnetic resonance imaging study of individual joints in patients with early rheumatoid arthritis. Arthritis Rheum.

[B25] Boers M, Verhoeven AC, Markusse HM, van de Laar MA, Westhovens R, van Denderen JC, van Zeben D, Dijkmans BA, Peeters AJ, Jacobs P (1997). Randomised comparison of combined step-down prednisolone, methotrexate and sulphasalazine with sulphasalazine alone in early rheumatoid arthritis. Lancet.

[B26] Bathon JM, Martin RW, Fleischmann RM, Tesser JR, Schiff MH, Keystone EC, Genovese MC, Wasko MC, Moreland LW, Weaver AL (2000). A comparison of etanercept and methotrexate in patients with early rheumatoid arthritis. N Engl J Med.

[B27] Visser H, le Cessie S, Vos K, Breedveld FC, Hazes JM (2002). How to diagnose rheumatoid arthritis early: a prediction model for persistent (erosive) arthritis. Arthritis Rheum.

[B28] Mottonen T, Hannonen P, Korpela M, Nissila M, Kautiainen H, Ilonen J, Laasonen L, Kaipiainen-Seppanen O, Franzen P, Helve T (2002). Delay to institution of therapy and induction of remission using single-drug or combination-disease-modifying antirheumatic drug therapy in early rheumatoid arthritis. Arthritis Rheum.

[B29] Kurki P, Aho K, Palosuo T, Heliovaara M (1992). Immunopathology of rheumatoid arthritis. Antikeratin antibodies precede the clinical disease. Arthritis Rheum.

[B30] Berglin E, Padyukov L, Sundin U, Hallmans G, Stenlund H, van Venrooij WJ, Klareskog L, Dahlqvist SR (2004). A combination of autoantibodies to cyclic citrullinated peptide (CCP) and HLA-DRB1 locus antigens is strongly associated with future onset of rheumatoid arthritis. Arthritis Res Ther.

[B31] Nielen MM, Van Schaardenburg D, Reesink HW, van de Stadt RJ, van der Horst-Bruinsma IE, de Koning MH, Habibuw MR, Vandenbroucke JP, Dijkmans BA (2004). Specific autoantibodies precede the symptoms of rheumatoid arthritis: a study of serial measurements in blood donors. Arthritis Rheum.

[B32] Bucht A, Larsson P, Weisbrot L, Thorne C, Pisa P, Smedegard G, Keystone EC, Gronberg A (1996). Expression of interferon-gamma (IFN-gamma), IL-10, IL-12 and transforming growth factor-beta (TGF-beta) mRNA in synovial fluid cells from patients in the early and late phases of rheumatoid arthritis (RA). Clin Exp Immunol.

[B33] Schumacher HR, Kitridou RC (1972). Synovitis of recent onset. A clinicopathologic study during the first month of disease. Arthritis Rheum.

[B34] Firestein GS (2003). Evolving concepts of rheumatoid arthritis. Nature.

[B35] Morita Y, Yamamura M, Kawashima M, Harada S, Tsuji K, Shibuya K, Maruyama K, Makino H (1998). Flow cytometric single-cell analysis of cytokine production by CD4^+ ^T cells in synovial tissue and peripheral blood from patients with rheumatoid arthritis. Arthritis Rheum.

[B36] Seo SK, Choi JH, Kim YH, Kang WJ, Park HY, Suh JH, Choi BK, Vinay DS, Kwon BS (2004). 4-1BB-mediated immunotherapy of rheumatoid arthritis. Nat Med.

[B37] Heinzel FP, Sadick MD, Mutha SS, Locksley RM (1991). Production of interferon gamma, interleukin 2, interleukin 4, and interleukin 10 by CD4^+ ^lymphocytes in vivo during healing and progressive murine leishmaniasis. Proc Natl Acad Sci USA.

[B38] Modlin RL (1994). Th1-Th2 paradigm: insights from leprosy. J Invest Dermatol.

[B39] Gerli R, Bistoni O, Russano A, Fiorucci S, Borgato L, Cesarotti ME, Lunardi C (2002). In vivo activated T cells in rheumatoid synovitis. Analysis of Th1- and Th2-type cytokine production at clonal level in different stages of disease. Clin Exp Immunol.

[B40] Joosten LA, Lubberts E, Helsen MM, Saxne T, Coenen-de Roo CJ, Heinegard D, van den Berg WB (1999). Protection against cartilage and bone destruction by systemic interleukin-4 treatment in established murine type II collagen-induced arthritis. Arthritis Res.

[B41] Jacobs MJ, van den Hoek AE, van Lent PL, van de Loo FA, van de Putte LB, van den Berg WB (1994). Role of IL-2 and IL-4 in exacerbations of murine antigen-induced arthritis. Immunology.

[B42] Jakubzick C, Choi ES, Kunkel SL, Joshi BH, Puri RK, Hogaboam CM (2003). Impact of interleukin-13 responsiveness on the synthetic and proliferative properties of Th1- and Th2-type pulmonary granuloma fibroblasts. Am J Pathol.

[B43] Kaufman J, Sime PJ, Phipps RP (2004). Expression of CD154 (CD40 ligand) by human lung fibroblasts: differential regulation by IFN-gamma and IL-13, and implications for fibrosis. J Immunol.

[B44] Chiaramonte MG, Donaldson DD, Cheever AW, Wynn TA (1999). An IL-13 inhibitor blocks the development of hepatic fibrosis during a T-helper type 2-dominated inflammatory response. J Clin Invest.

[B45] Relic B, Guicheux J, Mezin F, Lubberts E, Togninalli D, Garcia I, van den Berg WB, Guerne PA (2001). IL-4 and IL-13, but not IL-10, protect human synoviocytes from apoptosis. J Immunol.

[B46] Salmon M, Pilling D, Borthwick NJ, Akbar AN (1997). Inhibition of T cell apoptosis – a mechanism for persistence in chronic inflammation. The Immunologist.

[B47] Bucala R, Ritchlin C, Winchester R, Cerami A (1991). Constitutive production of inflammatory and mitogenic cytokines by rheumatoid synovial fibroblasts. J Exp Med.

[B48] Parsonage G, Falciani F, Burman A, Filer A, Ross E, Bofill M, Martin S, Salmon M, Buckley CD (2003). Global gene expression profiles in fibroblasts from synovial, skin and lymphoid tissue reveals distinct cytokine and chemokine expression patterns. Thromb Haemost.

[B49] Weyand CM, Goronzy JJ (2003). Ectopic germinal center formation in rheumatoid synovitis. Ann N Y Acad Sci.

[B50] Leung BP, Conacher M, Hunter D, McInnes IB, Liew FY, Brewer JM (2002). A novel dendritic cell-induced model of erosive inflammatory arthritis: distinct roles for dendritic cells in T cell activation and induction of local inflammation. J Immunol.

[B51] Zhou LJ, Tedder TF (1996). CD14^+ ^blood monocytes can differentiate into functionally mature CD83+ dendritic cells. Proc Natl Acad Sci USA.

[B52] Santiago-Schwarz F, Anand P, Liu S, Carsons SE (2001). Dendritic cells (DCs) in rheumatoid arthritis (RA): progenitor cells and soluble factors contained in RA synovial fluid yield a subset of myeloid DCs that preferentially activate Th1 inflammatory-type responses. J Immunol.

[B53] Blades MC, Ingegnoli F, Wheller SK, Manzo A, Wahid S, Panayi GS, Perretti M, Pitzalis C (2002). Stromal cell-derived factor 1 (CXCL12) induces monocyte migration into human synovium transplanted onto SCID Mice. Arthritis Rheum.

[B54] Miossec P (2003). Interleukin-17 in rheumatoid arthritis: if T cells were to contribute to inflammation and destruction through synergy. Arthritis Rheum.

[B55] Fossiez F, Djossou O, Chomarat P, Flores-Romo L, Ait-Yahia S, Maat C, Pin JJ, Garrone P, Garcia E, Saeland S (1996). T cell interleukin-17 induces stromal cells to produce proinflammatory and hematopoietic cytokines. J Exp Med.

[B56] Jovanovic DV, Di Battista JA, Martel-Pelletier J, Jolicoeur FC, He Y, Zhang M, Mineau F, Pelletier JP (1998). IL-17 stimulates the production and expression of proinflammatory cytokines, IL-beta and TNF-alpha, by human macrophages. J Immunol.

[B57] Akbar AN, Borthwick NJ, Wickremasinghe RG, Panayoitidis P, Pilling D, Bofill M, Krajewski S, Reed JC, Salmon M (1996). Interleukin-2 receptor common gamma-chain signaling cytokines regulate activated T cell apoptosis in response to growth factor withdrawal: selective induction of anti-apoptotic (bcl-2, bcl-xL) but not pro- apoptotic (bax, bcl-xS) gene expression. Eur J Immunol.

